# Label-free quantitative proteomic analysis of alfalfa in response to microRNA156 under high temperature

**DOI:** 10.1186/s12864-020-07161-1

**Published:** 2020-11-02

**Authors:** Muhammad Arshad, Alpa Puri, Aaron J. Simkovich, Justin Renaud, Margaret Y. Gruber, Frédéric Marsolais, Abdelali Hannoufa

**Affiliations:** 1 Agriculture and Agri-Food Canada, 1391 Sandford Street, London, Ontario N5V 4T3 Canada; 2 Centre for Genomics and Systems Biology, New York University, Abu Dhabi, United Arab Emirates; 3 Department of Biology, University of Western Ontario, 1151 Richmond Street, London, Ontario N6A 5B7 Canada; 4 Agriculture and Agri-Food Canada, 107 Science Place, Saskatoon, Saskatchewan S7N 0X2 Canada

**Keywords:** Alfalfa, Heat stress, miR156, Proteomic, LC-MS/MS

## Abstract

**Background:**

Abiotic stress, including heat, is one of the major factors that affect alfalfa growth and forage yield. The small RNA, microRNA156 (miR156), regulates multiple traits in alfalfa during abiotic stress. The aim of this study was to explore the role of miR156 in regulating heat response in alfalfa at the protein level.

**Results:**

In this study, we compared an empty vector control and miR156 overexpressing (miR156OE) alfalfa plants after exposing them to heat stress (40 °C) for 24 h. We measured physiological parameters of control and miR156OE plants under heat stress, and collected leaf samples for protein analysis. A higher proline and antioxidant contents were detected in miR156OE plants than in controls under heat stress. Protein samples were analyzed by label-free quantification proteomics. Across all samples, a total of 1878 protein groups were detected. Under heat stress, 45 protein groups in the empty vector plants were significantly altered (*P* < 0.05; |log_2_FC| > 2). Conversely, 105 protein groups were significantly altered when miR156OE alfalfa was subjected to heat stress, of which 91 were unique to miR156OE plants. The identified protein groups unique to miR156OE plants were related to diverse functions including metabolism, photosynthesis, stress-response and plant defenses. Furthermore, we identified transcription factors in miR156OE plants, which belonged to squamosa promoter binding-like protein, MYB, ethylene responsive factors, AP2 domain, ABA response element binding factor and bZIP families of transcription factors.

**Conclusions:**

These results suggest a positive role for miR156 in heat stress response in alfalfa. They reveal a miR156-regulated network of mechanisms at the protein level to modulate heat responses in alfalfa.

## Background

Alfalfa (*Medicago sativa* L.) is an important leguminous crop that is grown worldwide as forage for livestock feed, and contributes to improved soil quality. Another important feature of this crop is the potential for multiple harvests throughout the growing season, allowing for abundant biomass yield. Perennial nature of alfalfa and its rapid biomass production make it a suitable source for bioenergy purpose. However, these benefits are offset by alfalfa’s susceptibility to environmental stresses including heat, which exerts adverse effects on its growth and productivity [1]. High temperature can negatively affect plant growth and development including reduced seed germination [2], damage to leaves and branches, increase in leaf senescence, discoloring of fruits, which ultimately leads to poor crop yield [3]. Climate models have predicted an increase in seasonal temperatures globally, which may have a negative impact on crop growth, productivity and ultimately food security [4]. Developing alfalfa cultivars with improved heat stress tolerance could provide a sustainable solution to the unpredictable changes in the environmental conditions.

Molecular approaches have widely been used for dissecting the underlying biological and cellular processes under abiotic stress in plants [5–8]. Proteomic approaches have increasingly been used in plant research, and in particular to study abiotic stress responses as changes in protein abundance play a vital role in stress tolerance [9–12]. Proteomic analysis is a powerful technique to study gene products (proteins) at the molecular level [13, 14], and these have been used to study the underlying molecular and physiological processes for heat stress tolerance in different plant species. For example, a proteomic study showed that abundance of heat shock proteins (HSPs) and antioxidant enzymes were increased in heat-stressed leaves of rice [15]. Moreover, a heat stress-induced abundance of various protein groups involved in protein biosynthesis, degradation, and carbohydrate metabolism was reported in rice [16]. A similar study in grapevine showed that the abundance of HSPs and proteins involved in metabolism and signal transduction was significantly altered under heat stress [17]. Similarly, another study reported the differential abundance of 81 protein groups under heat stress in alfalfa. These proteins belonged to important functional categories such as metabolism, energy, protein synthesis, signal transduction and defense [6].

The microRNAs (miRNAs) are key regulators of gene expression at both the transcriptional and post-transcriptional levels [18, 19]. These miRNAs are approximately 18–24 nt long and are grouped based on the differences in their biogenesis and functional characteristics [20]. Recently, microRNA156 (miR156) has emerged as an effective molecular tool for trait improvement in different plant species including alfalfa. For example, miR156 overexpression increased alfalfa biomass and delayed flowering [21, 22]. Moreover, transcriptome analysis of miR156 overexpressing (miR156OE) alfalfa under drought revealed potential miR156 targets, and subsequent characterization confirmed its role in drought tolerance [23]. Major transcription factors regulated by miR156 belong to Squamosa Promoter Binding Protein-Like (SPL) family [24]. Previously, we identified miR156 target SPL genes, and characterized their functions in alfalfa, including their role in drought and salinity responses [25–27]. Despite a series of miR156-related studies in different plant species, there has been no reported proteome analysis on miR156OE alfalfa under heat stress. Proteome analysis of contrasting alfalfa genotypes under heat stress conditions could provide an insight into the underlying molecular mechanisms that control different physiological and molecular traits in alfalfa.

Accumulation of osmoprotectants, such as proline, is an important physiological mechanism that helps plants scavenge reactive oxygen species (ROS) to cope with heat-related oxidative stress [28, 29]. Proline helps plants keep a fully functional photosynthetic apparatus by stabilizing the photosynthetic complex II as well as membrane proteins such as rubisco [30]. Studies have shown that proline accumulation improves heat stress tolerance in a range of plant species such as tomato [31], rice [32], chickpea [33] and barley [34].

Recently, our group has shown that miR156 overexpression resulted in an improved physiological response of alfalfa to heat stress [35]. This finding triggered our interest in expanding our research to dissect the role of miR156 in modulating the proteome of alfalfa in response to heat. We employed a label-free quantification (LFQ) based quantitative proteomics approach to explore the effects of heat stress on protein levels in miR156OE alfalfa. Our major objective was to identify miR156-regulated gene products with differentially altered abundance under heat stress. In the current study, miR156OE plants showed enhanced levels of stress tolerance predictors (antioxidants and proline) under heat stress. Moreover, this study revealed that metabolism, photosynthesis and defense were the major processes affected by miR156 under heat. This combination of biochemical and proteomic analyses with miR156 influence provided additional knowledge of heat tolerance mechanisms, thereby shedding a light on the pathways mediated by miR156 for heat stress response in alfalfa.

## Results

Findings from our previous study that miR156 modulated heat stress response in alfalfa [35], prompted us to further study the molecular mechanism for regulation of heat stress tolerance. We conducted this research with an aim to identify proteins with differentially altered abundance modulated by miR156 under high temperature.

### Biochemical characterization of miR156OE alfalfa

**Fig. 1:**
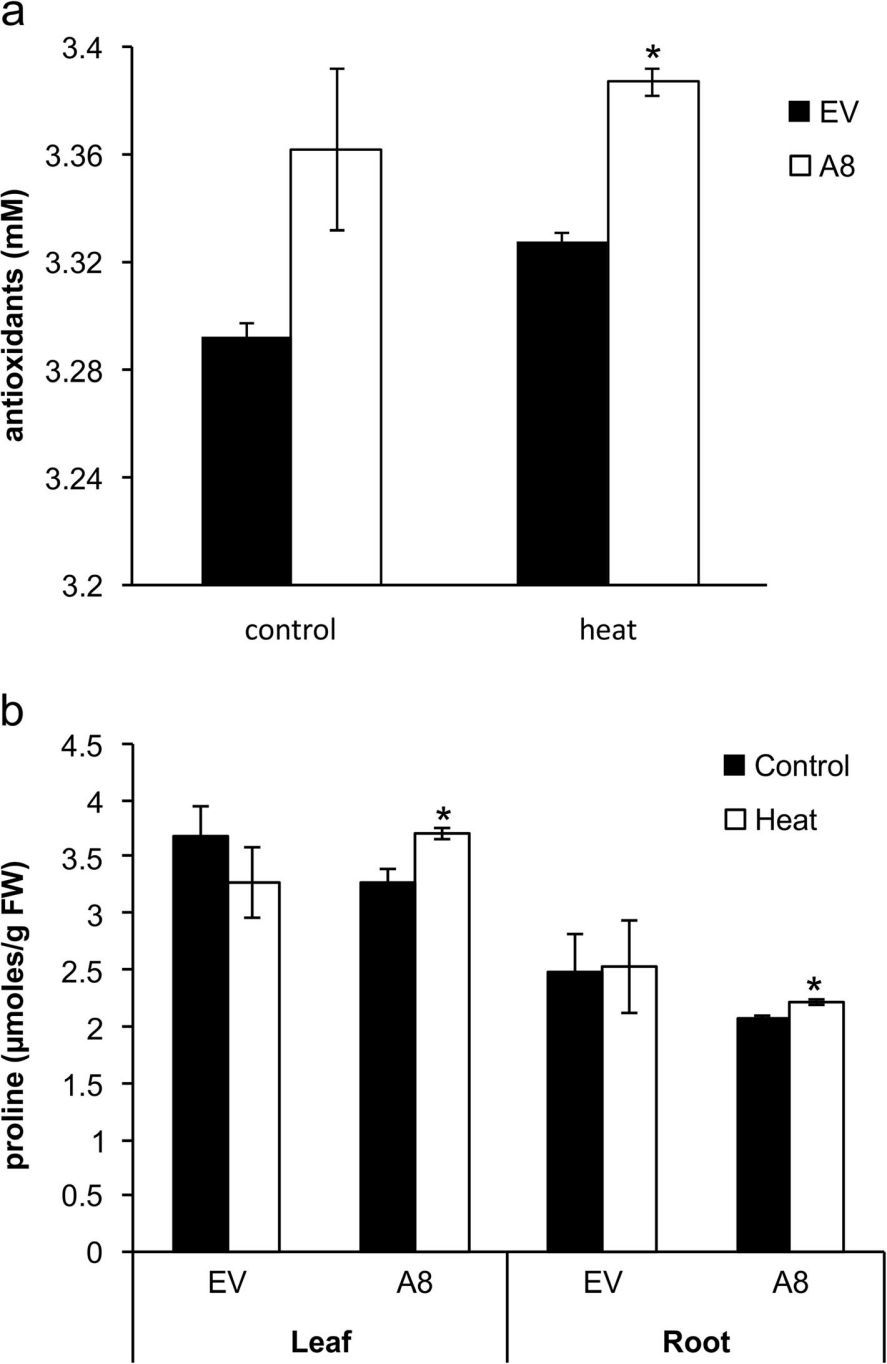
MiR156 alters the physiological responses of alfalfa to heat stress. Levels of **a** antioxidants, and **b** proline in EV control and A8. Asterisk (*) shows statistical significance at *p* < 0.05 where *n* = 3–4 (t-test)

Plants produce free radicals in response to stress, and these can be harmful to cellular membrane and lipids. To counter the negative effect of these free radicals, plants synthesize antioxidants as a defense mechanism, which prevent cellular damage by quenching free radicals [36]. To explore whether miR156 alters the ability of alfalfa to produce antioxidants for defense, we determined total antioxidant contents in EV and A8 under non-stressed control and heat stress conditions. Overall, A8 showed a mild increase in antioxidant content under non-stress control and stress conditions compared to EV (Fig. 1a).

As a defence mechanism, plants synthesize an array of metabolites under stress conditions including amino acids such as proline. Proline is a compatible solute that can help plants to increase water uptake from soil. Higher levels of proline are beneficial for plants under abiotic stress conditions [36]. In the current study, no significant difference in proline accumulation was observed between non-stressed and stressed plants for the EV control in either leaf or root (Fig. 1b). On the other hand, a significant increase in proline accumulation was detected in both heat-stressed leaf and root of A8 compared to corresponding non-stressed control plants (Fig. 1b).

### Alfalfa proteome is affected by miR156 under heat stress

**Fig. 2:**
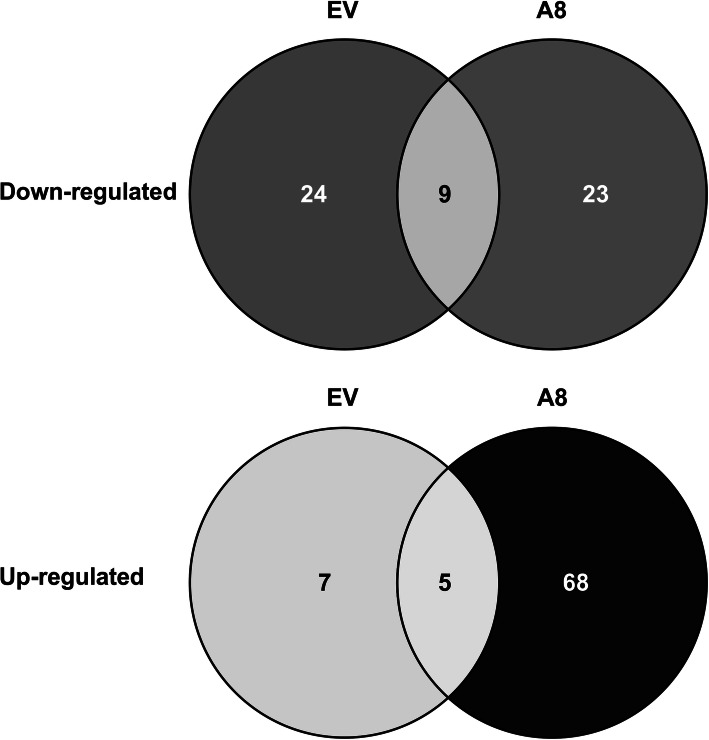
Comparison of protein with differential abundance in EV and A8. Venn diagram shows the number of significantly downregulated (**a**) and upregulated (**b**) proteins in EV and A8 under heat stress relative to corresponding non-stress controls

Previously, our group showed that overexpression of miR156 improved multiple physiological traits and altered the transcriptome profile of alfalfa [37]. Further investigations revealed a positive role for miR156 in abiotic stress tolerance, including drought [26, 38], salinity [27] and heat [35]. In addition, transcriptomic analysis showed that miR156 affects a wide array of gene families under drought stress in alfalfa [23]. We, therefore, set out to identify miR156-regulated gene products (proteins) with differentially altered abundance under heat stress in alfalfa. Across all samples, a total of 1878 protein groups were detected (online repository). To assess the effect of heat on the protein profile, protein abundance was compared between non-stressed control and heat-stressed plants of EV and A8. Results illustrated that the abundance of 12 proteins was significantly increased (*P* < 0.05; log2FC > 1), and that of 33 proteins was significantly decreased (P < 0.05; log2FC < − 1), in EV under heat stress relative to the corresponding non-stressed plants (Fig. 2a, b; Table 1). On the other hand, almost six-fold number of proteins (73) showed significantly enhanced abundance, and 32 proteins showed reduced abundance in A8 under heat stress relative to the corresponding A8 non-stressed plants (Fig. 2a, b; Table 2). While a small number of the differentially altered proteins (14) were common to both genotypes, a total of 91 proteins exhibited differential abundance uniquely in A8 under heat stress (68 proteins increased; 23 proteins reduced) (Fig. 2; Tables 1 and 2).

**Table 1:** Identified proteins with differentially altered abundance in EV controls under heat stress relative to the non-stress EV control plants

Protein ID	Locus name	Log2 (fold change)	FDR	annotation
G7JFK1	MTR_4g130540	−7.83	0.0161	Heat shock 70 kDa protein
Q2HTU2	MTR_4g091590	−7.00	0.0191	17.6 kDa class I heat shock protein
A0A072TL89	MTR_0004s051	−5.80	0.0035	Putative small heat shock protein HSP20
A0A072UP91	MTR_4g084250	− 4.83	0.0191	Calcyclin-binding protein
G7LF61	MTR_8g012340	−4.56	0.0275	Peptidylprolyl isomerase
G7L491	MTR_7g012820	−4.37	0.0191	Casein lytic proteinase B3
G7KD12	MTR_5g090410	−4.14	0.0002	Oxygen-evolving enhancer protein 2–1
G7KG40	MTR_5g078040	−3.56	0.0375	Peroxisomal small heat shock protein
G7L1Y9	MTR_7g010800	−3.45	0.0161	ATP-dependent zinc metalloprotease FTSH protein
G7KNT7	MTR_6g061940	−3.28	0.0436	17.6 kDa class I heat shock protein
G7J8C7	MTR_3g104780	−3.23	0.0462	17.1 kDa class II heat shock protein
G7K4W1	MTR_5g096970	−3.13	0.0035	Carboxy-terminal TIM barrel domain enolase
G7JMP4	MTR_4g104300	−3.10	0.0345	F-box/RNI/FBD-like domain protein
G7IRL3	MTR_2g089340	−2.95	0.0127	Dihydroxyacid dehydratase
G7KG90	MTR_5g012030	−2.86	0.0127	Putative Heat shock chaperonin-binding
A0A072VBG9	MTR_2g084715	−2.84	0.0018	Putative transcription factor C3H family
G7L4S2	MTR_7g088490	−2.77	0.0327	Proteasome subunit beta
A0A072UGC6	MTR_5g073235	−2.50	0.0190	Uncharacterized protein
G7KGT1	MTR_5g080450	−2.32	0.0115	Ribulose bisphosphate carboxylase/oxygenase activase
G7KW94	MTR_7g093500	−2.30	0.0277	Activator of 90 kDa heat shock ATPase-like protein
G7JL07	MTR_4g072110	−2.19	0.0191	Amidophosphoribosyltransferase
B7FLU4	MTR_4g103790	−1.99	0.0375	NOP56-like pre RNA processing ribonucleoprotein
A0A072TVH5	MTR_0009s039	−1.95	0.0434	Heat shock protein 81–2
G7KWU8	MTR_7g024390	−1.93	0.0126	Heat shock cognate 70 kDa protein
A0A072UQ41	MTR_4g105490	−1.60	0.0375	Synaptobrevin-like protein
G7J3Q2	MTR_3g087030	−1.50	0.0478	Molecular chaperone Hsp40/DnaJ family protein
G7KEN6	MTR_5g097320	−1.50	0.0115	Heat shock protein 81–2
G7JNG4	MTR_4g074480	−1.49	0.0399	Anamorsin homolog
G7IHD7	MTR_2g082590	−1.46	0.0345	Thioredoxin
B7FKA1	MTR_4g021570	−1.43	0.0191	Armadillo/beta-catenin-like repeat protein
A0A072VEG8	MTR_1g017380	−1.28	0.0035	Putative chaperonin Cpn60/TCP-1 family
G7I9Z6	MTR_1g031650	−1.26	0.0191	Calcium-dependent lipid-binding (CaLB domain) family
A0A072UL44	MTR_4g063710	−1.23	0.0044	Heat shock cognate 70 kDa protein
G7I6D7	MTR_1g011800	1.07	0.0184	Plant/F18G18–200 protein
A0A072TUF8	MTR_8g090025	1.55	0.0269	Haloacid dehalogenase-like hydrolase
G7ICF3	MTR_1g018510	2.52	0.0184	Subtilisin-like serine protease
G7ILM0	MTR_2g017730	2.52	0.0393	Heat shock 70 kDa protein
A0A072UMH4	MTR_4g066170	2.55	0.0191	Lipoxygenase
G7JCT4	MTR_4g095360	2.79	0.0351	Putative tripeptidyl-peptidase II
A0A072VFH5	MTR_2g023540	2.98	0.0253	5-adenylylsulfate reductase
G7IAX3	MTR_1g116270	3.19	0.0359	Glutathione S-transferase
A0A072UZV5	MTR_3g078633	3.63	0.0191	Enhanced disease susceptibility protein
A0A072U496	MTR_7g113480	4.25	0.0044	Xaa-pro aminopeptidase P
G7J6G6	MTR_3g116110	4.34	0.0184	Photosystem II reaction center PsbP family protein
B7FKA0	MTR_5g035010	4.62	0.0115	Polyketide cyclase/dehydrase and lipid transporter

**Table 2:** Identified proteins with differentially altered abundance in miR156 overexpressing genotype (A8) under heat stress relative to the non-stress control A8 plants

Protein IDs	Locus name	Log2 (fold change)	FDR	annotation
^a^G7JFK1	MTR_4g130540	−7.85	0.0064	Heat shock 70 kDa protein
A0A072U9J1	MTR_6g452990	−6.82	0.0080	Heat shock protein 81–2
^a^A0A072TL89	MTR_0004s051	−6.52	0.0207	Putative small heat shock protein HSP20
^a^G7KG40	MTR_5g078040	−4.57	0.0050	Peroxisomal small heat shock protein
G7JGX6	MTR_4g010130	−4.40	0.0157	Sterol regulatory element-binding protein
G7IF74	MTR_1g088640	−4.31	0.0048	Putative universal stress protein A
^a^G7IRL3	MTR_2g089340	−3.60	0.0076	Dihydroxyacid dehydratase
G7LGJ8	MTR_8g095680	−3.33	0.0173	Calnexin 2
^a^G7KWU8	MTR_7g024390	−3.24	0.0160	Heat shock cognate 70 kDa protein
^a^G7L491	MTR_7g012820	−2.70	0.0080	Casein lytic proteinase B3
G7K8X5	MTR_5g059210	−2.63	0.0340	Ubiquitin-fold modifier 1
G7JM88	MTR_4g057200	−2.54	0.0048	Lethal leaf-spot protein, putative
G7K9T0	MTR_5g038460	−2.41	0.0204	Plant/T7N9–9 protein
A0A072UC14	MTR_7g077400	−2.29	0.0058	Acyl-CoA thioesterase
G7JI82	MTR_3g082660	−2.06	0.0076	Bacterial long-chain fatty acid CoA synthetase
^a^G7KEN6	MTR_5g097320	−1.89	0.0258	Heat shock protein 81–2
G7IQD5	MTR_2g045050	−1.86	0.0209	Acyl-CoA thioesterase
G7L9N5	MTR_8g089560	−1.76	0.0380	Putative RIN4, pathogenic type III effector
A2Q5W0	MTR_7g085800	−1.68	0.0422	Tubulin alpha chain
^a^B7FKA1	MTR_4g021570	−1.58	0.0350	Armadillo/beta-catenin-like repeat protein
G7JNV8	MTR_4g106880	−1.55	0.0069	Peroxisomal membrane PEX14-like protein
G7IMW8	MTR_2g034900	−1.51	0.0076	Importin subunit alpha
G7JNZ5	MTR_4g122670	−1.38	0.0105	Mevalonate/galactokinase family protein
^a^A0A072UL44	MTR_4g063710	−1.29	0.0173	Heat shock cognate 70 kDa protein
G7L8K5	MTR_8g046300	−1.20	0.0258	Prohibitin
G7JRF5	MTR_4g036260	−1.19	0.0202	Quinone-oxidoreductase-like protein
G7K595	MTR_5g016590	−1.18	0.0301	Proteasome subunit alpha type
A0A072TUS4	MTR_8g099795	−1.16	0.0292	Heat shock 70 kDa protein
G7I7Q4	MTR_1g025430	−1.15	0.0173	Heat shock protein 81–2
G7I836	MTR_1g082870	−1.10	0.0173	Mitochondrial Rho GTPase
G7LIP6	MTR_8g086070	−1.03	0.0341	Dicarboxylate carrier protein
A0A072VXV5	MTR_1g077480	−1.00	0.0392	Alpha-galactosidase
G7KUS5	MTR_7g022440	1.03	0.0329	Glucose-6-phosphate 1-dehydrogenase
G7JW95	MTR_5g022300	1.07	0.0155	Ferredoxin--NADP reductase, chloroplastic
B7FJJ4	MTR_7g005380	1.12	0.0269	Pyruvate dehydrogenase E1 component subunit
G7JAP0	MTR_3g070100	1.15	0.0337	Putative sedoheptulose-bisphosphatase
G7JI05	MTR_4g131760	1.28	0.0096	Glucose-1-phosphate adenylyltransferase
Q45FF2	MTR_2g017520	1.29	0.0080	Q45FF2_MEDTR Pyridoxal 5-phosphate synthase
A2Q5N9	MTR_7g085490	1.29	0.0155	Galactose mutarotase-like
G7IBQ7	MTR_1g086050	1.31	0.0429	Protein translocase subunit SecA
G7K882	MTR_5g027530	1.31	0.0080	Phosphoribulokinase
Q84UC1	MTR_2g021255	1.32	0.0274	Glutamine synthetase
A0A072V8Q4	MTR_2g046710	1.32	0.0144	S-adenosylmethionine synthase
G7IED1	MTR_1g072260	1.36	0.0221	Putative NAD(P)-binding domain-containing
A0A072UUQ2	MTR_4g045980	1.39	0.0173	Photosystem II biogenesis protein
A0A072ULB0	MTR_4g071880	1.39	0.0203	Fructose-bisphosphate aldolase
A0A072UDY2	MTR_5g004680	1.40	0.0173	Presequence protease
A0A072VPY5	MTR_1g023120	1.41	0.0389	Beta-galactosidase
A0A072V4D0	MTR_3g112420	1.42	0.0392	ATP-dependent protease LA (Lon) domain protein
G7J8Z9	MTR_3g092720	1.44	0.0329	Putative ribosomal protein S30Ae/sigma
G7L028	MTR_7g026340	1.46	0.0114	Glucan endo-1,3-beta-glucosidase-like protein
A0A072VMH0	MTR_1g076570	1.47	0.0173	2-methyl-6-phytylbenzoquinone methyltransferase
A0A072V2V0	MTR_3g498725	1.49	0.0185	ATP-dependent Clp protease ATP-binding subunit
A0A072U1Q8	MTR_7g066120	1.54	0.0188	Fructose-1,6-bisphosphatase
A0A072TGR0	MTR_0151s003	1.55	0.0444	Inositol-1-monophosphatase
A9YWS0	MTR_5g030950	1.56	0.0294	Serine hydroxymethyltransferase
I3S8V0	MTR_7g111860	1.57	0.0050	Putative NAD(P)-binding domain-containing protein
G7LJD5	MTR_8g070530	1.62	0.0105	Phototropin-2 protein
G7LE33	MTR_8g093770	1.65	0.0105	40S ribosomal protein S12
A0A072UYT5	MTR_3g068030	1.68	0.0202	Ribulose bisphosphate carboxylase/oxygenase activase
G7LIX6	MTR_8g018510	1.69	0.0086	Lipoxygenase
A0A072TX52	MTR_8g012565	1.73	0.0050	1-deoxy-D-xylulose 5-phosphate reductoisomerase
^a^G7JCT4	MTR_4g095360	1.76	0.0130	Putative tripeptidyl-peptidase II
G7L4Q1	MTR_7g077880	1.76	0.0258	Putative HAD-like domain-containing protein
G7K1Y1	MTR_5g079460	1.80	0.0173	PfkB family carbohydrate kinase
G7KG86	MTR_5g011990	1.82	0.0155	Uncharacterized protein
G7KET9	MTR_5g011220	1.84	0.0080	PGR5-like protein 1A
G7I2N9	MTR_1g073130	1.85	0.0479	Carboxy-terminal processing peptidase-like protein
G7K4T4	MTR_5g096670	1.87	0.0258	Fructose-bisphosphate aldolase
G7LA76	MTR_8g074330	1.88	0.0130	Chitinase (Class Ib) / Hevein
A0A072VNF5	MTR_1g096240	1.90	0.0405	Dihydrolipoamide acetyltransferase
A0A072UL99	MTR_4g071190	1.91	0.0202	Uncharacterized protein
G7JBK8	MTR_3g096290	1.91	0.0317	Cyanobacterial and plant NDH-1 subunit O
G7K999	MTR_5g009010	1.92	0.0117	Putative THUMP domain-containing protein
G7LH37	MTR_8g083210	1.95	0.0302	Aspartokinase-homoserine dehydrogenase
A0A072TEN7	MTR_0380s004	1.98	0.0173	Putative nucleoid-associated protein YbaB/EbfC
A0A072VAN9	MTR_2g090200	1.99	0.0048	Photosystem II Pbs27 protein
A0A072VRL6	MTR_1g107340	2.02	0.0156	Limonoid UDP glucosyltransferase, putative
A0A072UYV0	MTR_4g088615	2.03	0.0076	Putative ribosomal protein S5
I3SSE5	MTR_8g005175	2.04	0.0072	Oxygen-evolving enhancer protein
G7JK55	MTR_4g101750	2.06	0.0290	Elongation factor G, chloroplastic
G7KDR6	MTR_5g030020	2.09	0.0301	Putative nucleotide-binding alpha-beta protein
A0A072TTP2	MTR_8g080230	2.12	0.0166	Lipoxygenase
A0A072TLC8	MTR_0003s056	2.16	0.0340	Carboxypeptidase
G7JBQ7	MTR_3g108040	2.19	0.0080	PsbP domain protein
G7JEX7	MTR_4g068280	2.21	0.0048	Putative trigger factor
G7JZK0	MTR_5g071360	2.25	0.0173	Asparagine synthetase [glutamine-hydrolyzing]
A0A072VDJ3	MTR_2g105480	2.38	0.0130	Putative ATPase, AAA-type, P-loop
A0A072UF41	MTR_5g084030	2.39	0.0144	Indole-3-glycerol phosphate synthase
G7JFL4	MTR_4g130680	2.39	0.0329	ATP phosphoribosyltransferase catalytic subunit
G7KEX7	MTR_5g020640	2.47	0.0050	Glucose-6-phosphate 1-epimerase
G7LAE7	MTR_8g091410	2.51	0.0189	Peptidylprolyl isomerase
^a^A0A072UMH4	MTR_4g066170	2.53	0.0033	Lipoxygenase
G7J557	MTR_3g100500	2.70	0.0144	Aspartic proteinase nepenthesin-like protein
G7KPU0	MTR_6g088270	2.70	0.0207	Elongation factor Ts, mitochondrial
G8A394	MTR_3g073860	2.74	0.0290	Acetyl-CoA carboxylase
A0A072U5I5	MTR_7g117430	3.20	0.0064	Eukaryotic aspartyl protease family protein
G7L4R0	MTR_7g088340	3.23	0.0155	Magnesium-protoporphyrin IX monomethyl ester
^a^A0A072U496	MTR_7g113480	3.26	0.0050	Xaa-pro aminopeptidase P
A0A072VID5	MTR_1g052535	3.27	0.0270	GTP-binding protein TypA/BipA
A0A072UMH6	MTR_6g085010	3.55	0.0096	Aspartic protease in GUARD CELL-like protein
^a^G7J6G6	MTR_3g116110	3.98	0.0110	Photosystem II reaction center PsbP family protein
G7LAD5	MTR_8g091320	4.13	0.0050	Myo-inositol 1-phosphate synthase
^a^B7FKA0	MTR_5g035010	5.32	0.0080	Polyketide cyclase/dehydrase and lipid transporter
A0A072VIV9	MTR_1g052165	7.39	0.0219	Esterase D, putative

^a^Proteins that were common to A8 and EV genotypes under heat shock. All other proteins were unique to A8

*FDR* False discovery rate

The abundance of some proteins belonging to heat shock family was altered in both EV and A8 genotypes. Glutamine synthetase, fructose-bisphosphate aldolase (FBA), photosystem II proteins and Glucose-6-phosphate 1-dehydrogenase (G-6-PDH), Calnexin, lethal leaf-spot protein, α-galactosidase, β-galactosidase and Chitinase were among the major protein groups with known functions in plant stress response, and their abundance was differentially altered in A8 under heat stress (Table 2). These protein groups with altered abundance uniquely in miR156OE plants under heat stress (Table 2) could be potentially regulated by miR156 specifically under stress conditions.

### Gene ontology (GO) enrichment analysis

**Fig. 3:**
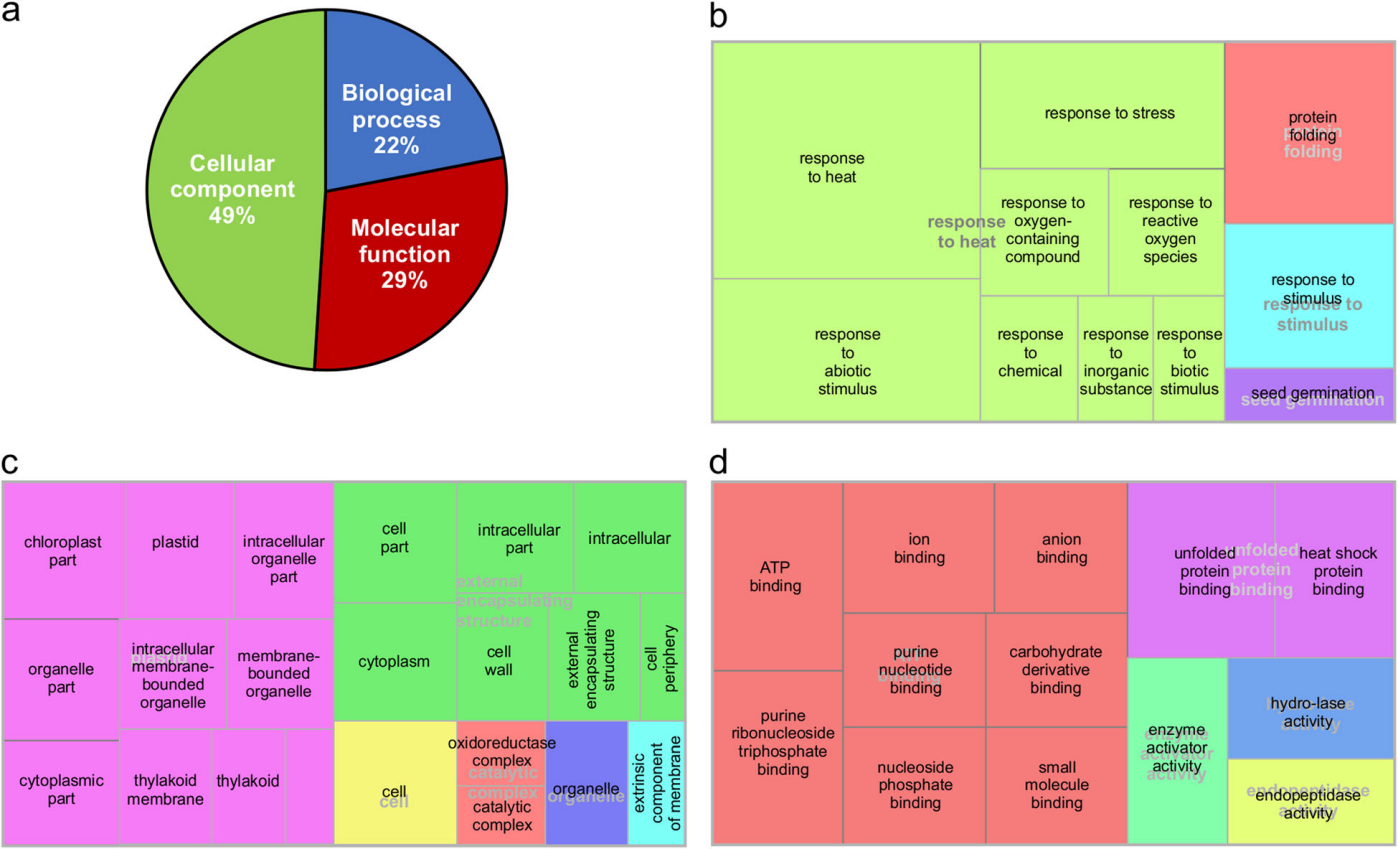
Gene Ontology (GO) enrichment analysis, **a** Fractional distribution of GO terms based on molecular function, cellular component and biological process. Tree maps of **b** biological process, **c** cellular component and **d** molecular function of identified proteins with differentially altered abundance in EV controls under heat stress

Gene ontology enrichment analysis was performed to identify pathways that may be affected in miR156OE plants under heat stress. We observed a large difference in GO function category representation between EV and miR156 genotypes. There were 76 GO terms that were assigned to protein with altered abundance in EV under stress, of which about half (49%) were represented by proteins belonging to the cellular component category (Fig. 3a). Only four, six and five GO functional categories were detected in the biological process, cellular component and molecular function categories, respectively. Biological process included response to heat, protein folding, response to stimulus and seed germination (Fig. 3b). Plastid, external encapsulating structure, cell, catalytic complex, organelle and extrinsic component of membrane were present in the cellular component category (Fig. 3c). Moreover, ATP binding, unfolded protein binding, enzyme activator activity, hydrolase activity and endopeptidase activity represented the molecular function category (Fig. 3d).

**Fig. 4:**
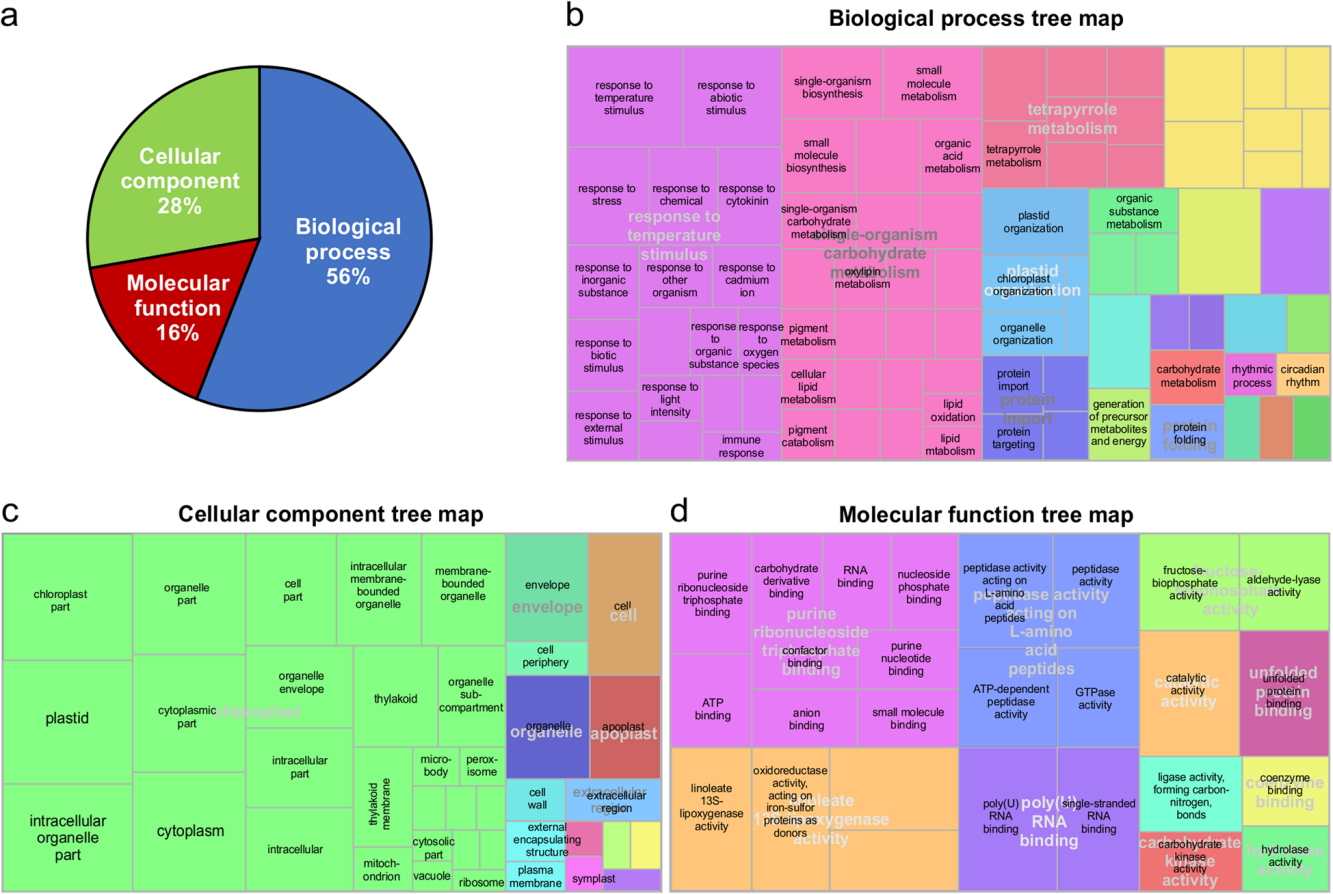
Gene Ontology (GO) enrichment analysis, **a** Fractional distribution of GO terms based on molecular function, cellular component and biological process. Tree maps of **b** biological process, **c** cellular component and **d** molecular function of identified proteins with differentially altered abundance in miR156 overexpressing genotype (A8) under heat stress

On the other hand, 227 GO terms were assigned to proteins with altered abundance in miR156 overexpressing genotype A8, of which a larger portion was represented by biological process (Fig. 4a). Many of these GO terms may reflect traits that miR156 overexpression modulates under stress conditions. Of the 21 GO terms in the biological process; response to temperature stimulus, single-organism carbohydrate metabolism, plastid organization and coenzyme metabolism (Fig. 4b) were unique to miR156 overexpression and may be of particular interest for stress response. The function chloroplast made up one of the largest portions in the cellular component category (Fig. 4c). In addition, extracellular region and apoplast were also represented by this category (Fig. 4c). Among the 11 functions classified as molecular function; purine ribonucleoside triphosphate binding, catalytic activity and fructose-bisphosphate adolase activity (Fig. 4d) were the main terms unique to miR156.

### Transcription factor enrichment

Transcription factor (TF) enrichment analysis was carried out for the 91 proteins unique to miR156 to explore the functional mechanism of miR156 transcriptional regulatory systems. A total of 37 TFs were predicted (Table 3) that may be affected by miR156 under heat stress conditions. Major transcription factor families included heat shock transcription factors, MYB transcription factors, ethylene responsive factor, TCP family transcription factor, squamosa promoter-binding-like protein (SPL), ABA response element-binding factor and bZIP transcription factor (Table 3).

**Table 3:** TF enrichment analysis showing TF families affected by specifically miR156 under heat stress conditions

Transcription factor	Annotation
Medtr6g086805	heat shock transcription factor
Medtr7g091370	heat shock transcription factor
Medtr4g022370	Dof domain zinc finger protein
Medtr3g077750	Dof domain zinc finger protein
Medtr8g005960	squamosa promoter-binding-like protein
Medtr2g099610	MYB transcription factor MYB91
Medtr2g043050	ethylene-responsive transcription factor ERF017-like protein
Medtr5g016750	ethylene response factor
Medtr4g111975	MYB-like transcription factor family protein
Medtr4g119270	ethylene response factor
Medtr7g015010	TCP family transcription factor
Medtr2g067420	myb transcription factor
Medtr7g010210	R2R3-myb transcription factor
Medtr4g100630	MYB-like transcription factor family protein
Medtr6g092540	MYB-like transcription factor family protein
Medtr7g067080	MYB transcription factor MYB51
Medtr7g083700	B3 domain transcription factor
Medtr7g080460	AP2-like ethylene-responsive transcription factor
Medtr4g108370	TCP family transcription factor
Medtr1g084980	phytochrome-interacting factor 3.1
Medtr6g017055	TCP family transcription factor
Medtr8g033250	MADS-box transcription factor
Medtr1g102860	heat shock transcription factor A3
Medtr3g101870	heat shock transcription factor
Medtr5g010680	heat shock transcription factor B2A
Medtr5g082950	AP2 domain class transcription factor
Medtr1g101810	TCP family transcription factor
Medtr7g028160	TCP family transcription factor
Medtr8g033070	TCP family transcription factor
Medtr5g026210	beta-amylase-like protein
Medtr1g062940	myb transcription factor
Medtr1g080920	transcription factor bZIP88
Medtr7g104480	ABA response element-binding factor
Medtr8g070820	bZIP transcription factor family protein
Medtr1g022495	BZIP transcription factor bZIP124
Medtr4g070860	BZIP transcription factor bZIP124
Medtr7g029400	BZIP transcription factor

## Discussion

Current climate change models predict an increase in average surface temperatures of 3 °C to 5 °C in the next 5 to 10 decades. This may have deleterious effects on crop plant growth and productivity [39]. High temperature can cause devastating effects on various aspects of plant function and physiology as well as disruption of cellular homeostasis [40]. Our group has recently shown that heat stress exerted negative impact on alfalfa plants where EV control leaves looked droopy and brownish whereas miR156 overexpression plants (miR156OE) including A8 maintained green and normal phenotype [35]. Moreover, miR156OE plants showed increased accumulation of antioxidants and water potential under heat stress compared to control plants. These results provided evidence that overexpression of miR156 enhances alfalfa tolerance to heat stress [35].

In the current study, MaxLFQ algorithm was used to assemble protein abundance profiles with maximum possible information from MS signals [41]. Heat stress response of miR156OE alfalfa was compared with that of the empty vector EV control genotype in an attempt to identify heat stress-related proteins regulated by miR156, as well as to further elucidate the biochemical and molecular mechanisms of heat tolerance in alfalfa, which are discussed below.

### Physiological response of miR156OE alfalfa to heat stress

High temperature can cause an array of physiological and biochemical changes in plants that adversely affect growth, development, and yield [40]. Plants have, however, evolved mechanisms to cope with environmental stressors. In response to heat stress, plants produce reactive oxygen species (ROS), which can serve as stress signals to trigger defense responses; at the same time, ROS can cause cellular damage [42]. To neutralize ROS, plants synthesize antioxidants that protect the cellular machinery by scavenging ROS [36]. Enhanced accumulation of antioxidants positively correlates with stress tolerance in several plant species [26, 27, 36]. Previously, our group showed that miR156OE alfalfa accumulated increased levels of antioxidants under drought and saline conditions, and the plants exhibited resilience to these stresses [26, 27]. In the current study, the miR156OE plants exhibited improved antioxidant capacity, which may suggest that miR156 can exert a defense response against ROS under heat stress conditions, and this could potentially improve heat stress tolerance in alfalfa.

Elevated proline levels help plants cope with stress, and accumulation of proline indicates improved cellular metabolism and enzymatic activity [43]. In line with the previous study that was conducted on non-transgenic control alfalfa plants [5], our results showed a mild increase in proline accumulation in leaf and root of miR156OE alfalfa, suggesting that miR156 may regulate the biosynthesis of this osmolyte in response to heat stress. In addition to affecting heat stress responses, we previously showed that genotype A8 accumulated higher proline and had elevated relative water content (RWC) under drought stress, and this genotype also displayed improved tolerance under this stress [26]. Together, these results support our current results that miR156 modulates a wide variety of abiotic stresses including heat [25–27, 44]. Increased proline accumulation correlates with higher RWC. A wheat genotype sensitive to drought exhibited reduced RWC at 30% of soil moisture, whereas RWC was not reduced in a drought tolerant genotype [45]. Similarly, a reduction in leaf water potential, stomatal conductance and transpiration rate, and an increase in leaf temperature and abscisic acid (ABA) level were observed in two genotypes of soybean under heat stress [46]. ABA is a stress hormone that triggers proline synthesis and helps plants combat stress conditions by altering physiological and molecular responses [47]. It will, therefore, be interesting to find out how miR156 modulates these physiological traits and hormone biosynthesis particularly ABA under heat stress.

### Functional processes affected by miR156 under heat stress

MicroRNAs have emerged as a vital component of post-transcriptional regulation of genes involved in numerous growth, development and stress responses in plants. The inhibitory effect of abiotic stress on photosynthesis is mainly linked to stomatal conductivity and metabolic limitations that have widely been described in several other studies, including studies on heat shock response [19, 48, 49]. In our current study, a number of proteins with altered abundance became prominent when heat stress was imposed on controls and miR156OE plants. Although, the number of proteins with reduced abundance in miR156 during heat stress was similar to that of the control, the number of proteins with increased abundance was six times more than the controls. This suggests that miR156 may be activating proteins for various physiological processes to cope with heat stress conditions. Interestingly, there were only 10% proteins common between control and miR156OE genotype whose abundance was altered under heat, indicating that miR156 may be modulating abundance of several unique proteins under the stress. In the current study, miR156OE alfalfa proteins responded to heat stress by modifying physiological processes that represent major protein groups under heat stress.

#### Photosynthesis

A large portion of cellular component GO term in miR156, but not in control, consists of chloroplast, indicating that photosynthetic processes are being modulated by miR156. Interestingly, our recent publication has shown that miR156OE alfalfa exhibited increased chlorophyll content under heat stress in alfalfa [35], which supports the proteomic response of miR156OE alfalfa in the current study. Photosynthesis is one of the major processes affected by abiotic stress [36], and energy deficit is a common indicator of photosynthetic plants under stress [50]. Overall, stress reduces photosynthesis and respiration, which leads to energy deprivation and ultimately growth retardation and cell death [50]. PSII is a sensitive protein complex and its structure is altered under abiotic stress [51]. Some heat shock proteins (HSPs) are involved in protecting PSII under heat stress [15, 16, 52]. A previous study in alfalfa showed 23 proteins with altered abundance under heat stress, and these proteins belonged to the PSII and HSPs [5].

An increased abundance of the photosynthetic enzyme fructose-bisphosphate aldolase (FBA) during stress maintains the CO_2_ assimilation rate in alfalfa [5]. Enhanced FBA abundance specifically in miR156 genotype under heat stress highlights the role of miR156 in altering the abundance of these proteins and maintaining photosynthesis under high temperature in alfalfa. Some other photosynthesis-related proteins with enhanced abundance were also detected in this study, including the oxygen evolving enhancer protein (OEE). Abiotic stress, such as cold and heat, alter the abundance of OEE family in plants [53]. In several plant species, this protein abundance was altered under abiotic stress [54], and in the current study OEE abundance was increased specifically in miR156OE genotype upon heat treatment. This suggests that OEE may directly or indirectly be regulated by miR156 and contributes to stress tolerance in alfalfa.

#### Metabolism

Plants allocate a significant supply of C and N resources to the synthesis of metabolites under stress conditions to maintain adequate growth [55]. Increased metabolic activity may be a vital response to elevated temperature. A reduction in photosynthesis results in energy shortage, which leads to the enhancement of carbohydrate metabolism. Previous studies have shown enhanced expression of glutamine synthetase (GS) under abiotic stress conditions [56]. In the current study, increased GS abundance specifically in miR156 genotype under heat stress may indicate that miR156 regulates *GS* expression. GS plays a crucial role in ammonia assimilation, and increased expression of cytosolic *GS* enhanced photorespiration and contributed to photosynthesis protection under stress condition [57].

Our results showed an increased abundance of other proteins (e.g. G-6-PDH, Calnexin, beta-galactosidase and Chitinase) that were previously reported to play a role in abiotic stress tolerance in various plant species. For example, transgenic tobacco overexpressing two chitinases (CHIT33 and CHIT42) conferred tolerance to salinity and heavy metals without any detrimental effect on plant growth and development [58]. Calnexin (CNX) maintains calcium homeostasis in plants and overexpression of CNX in tobacco improved tolerance to dehydration and osmotic stress [59]. Overexpression of β-galactosidase enhanced stress tolerance in Arabidopsis by increasing leaf area and reducing senescence [60], and we also observed an increased abundance of β-galactosidase in miR156OE plants under heat stress. Moreover, our study revealed a reduced α-galactosidase abundance in alfalfa under stress conditions, and these results are consistent with the previous research that showed down-regulation of α-galactosidase and ultimately improved tolerance to low temperature in petunia [61]. These observations suggest that miR156 modulates heat stress response in alfalfa by regulating some important proteins involved in physiological and metabolic processes.

#### Defense

Heat shock proteins (HSPs) are low molecular weight chaperones that play a vital role in providing plants with protection against stress by re-establishing normal protein conformation and cellular homeostasis, as well as assisting in protein refolding under stress. Li et al. (2013) detected 19 alfalfa proteins that belonged to the HSP group, most of which showed increased abundance in response to heat stress in alfalfa [5]. In contrast, a decrease in abundance of all HSPs (except one) and small heat shock protein (sHSP) was detected under heat stress in both control and miR156 genotypes. Plants induce expression of HSPs as an adaptive strategy for tolerance to heat stress. There are however substantial variations of HSP expression patterns in different plant species and even between genotypes of the same species [62]. Expression of four rice *HSPs* was rapidly increased under heat stress but two HSPs showed reduced expression after 3 h of heat stress in the same study, indicating that different *HSPs* were regulated by different time patterns or by different signals and may be affiliated with different functions in response to heat [62]. A repressive function of HSPs in this study is consistent with the finding that reduced HSP levels stimulated growth in *Arabidopsis* [63]. These differential responses by HSPs are of particular interest in the study of thermotolerance reactions in plants [15, 63] and need to be further investigated.

The small HSPs are of particular interest since they appear to protect PS II and thylakoid membranes under heat stress in plants [64]. Two studies have demonstrated the role of sHSPs in protecting the photosynthesis machinery. For example, sHSP interacts with proteins of the thermolabile oxygen-evolving complex (OEC) of PS II in *Chenopodium album* [65]. Similarly, an increase in sHSP26 abundance was found to improve the photochemical efficiency of PS II under heat stress in tall fescue [66]. These observations suggest that sHSPs can alter OEC proteins of PS II, pinpointing an important role for sHSPs in modulating plant response under high temperature. Although sHSPs may play a substantial role in protecting photosynthetic proteins against stress, more research is still needed to understand the underlying mechanisms governing the regulation of their biosynthesis and physiological functions, including their role in heat tolerance in plants under the influence of miR156.

Environmental stress, including high temperature, causes a rapid and excessive accumulation of reactive oxygen species (ROS) in plants. Excessive levels of stress-induced ROS are removed by enzymatic and non-enzymatic antioxidants [36]. This study showed an increased abundance of G-6-PHD and CNX in miR156OE plants under heat stress, and this is consistent with previous studies, which have shed light on the role of CNX in ROS signaling, scavenging ROS and improving oxidative stress response in plants [59, 67]. Similarly, Liu et al. (2007) revealed that G-6-PDH plays a crucial role in nitric oxide-dependant defence against oxidative stress, resulting in improved salt tolerance in red kidney beans [67].

### MicroRNA156 affects various transcription factors under heat stress

Transcription factors (TFs) play a crucial role in regulating molecular response under abiotic stress in plants. In the current study, we detected TCP, bZIP, ethylene responsive factor (ERF) and *SQUAMOSA-PROMOTER BINDING PROTEIN-LIKE* (SPL) by TF enrichment analysis, and our previous study showed an altered expression of these TFs under drought stress in miR156OE alfalfa [23]. This may indicate that miR156 regulates these TFs not only under drought but also heat stress conditions. The SPLs are known targets of miR156, and our recent studies have shown that reduced SPL13 expression improved drought [25] and heat [35] stress tolerance in alfalfa. Given the diversity of important TFs targeted by miR156, and the physiological traits affected by miR156 in alfalfa, it is critical to identify and characterize these TFs and their downstream targets to further elucidate the role of miR156-regulated network in stress tolerance.

## Conclusion

**Fig. 5:**
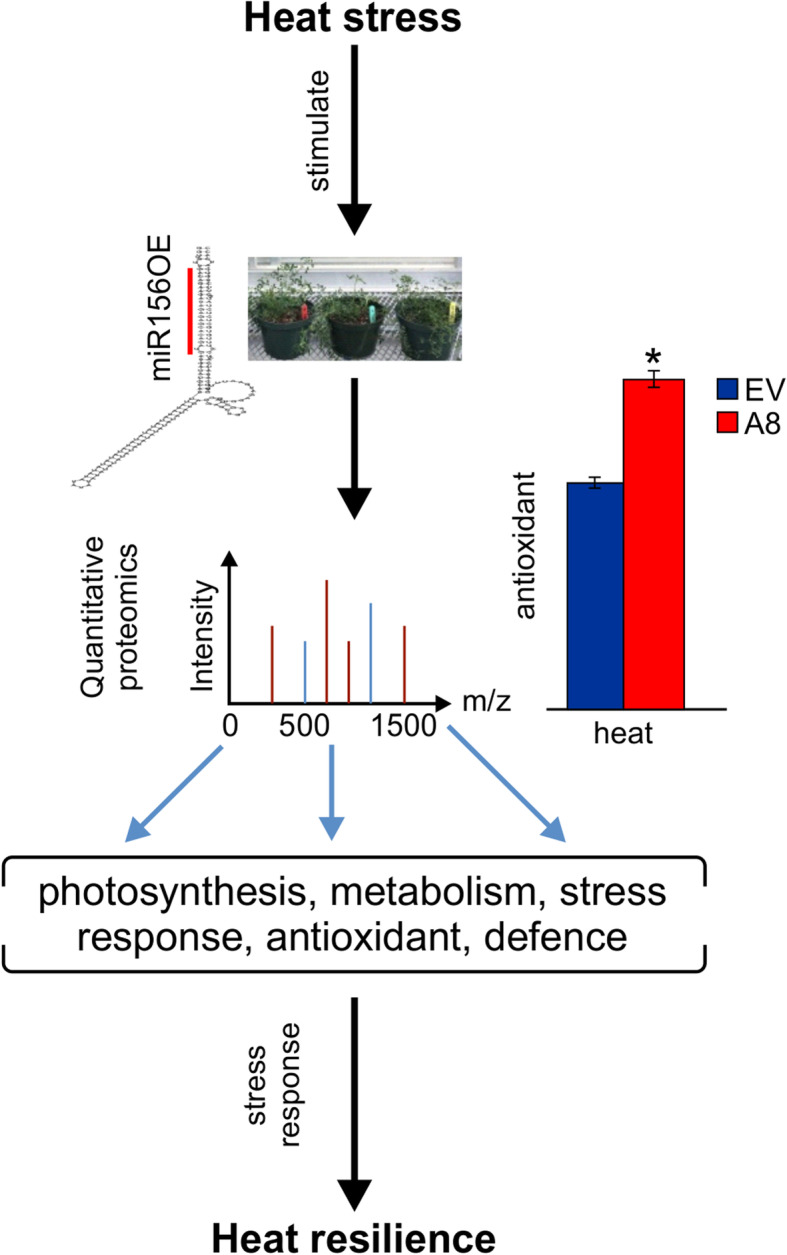
A proposed model of miR156-regulated heat stress response in alfalfa

In this study, we conducted label-free quantitative proteomics analysis on miR156OE alfalfa under heat stress. Our biochemical data showed that miR156OE plants accumulated higher levels of proline and antioxidants when exposed to elevated temperature (Fig. 5). Furthermore, LC-MS/MS analysis revealed differential abundance of a range protein groups in miR156OE plants under heat stress. We detected 91 proteins that were unique to miR156OE (undetected in EV alfalfa) and belong to critical functional groups such as plant defence, photosynthesis and metabolism. These proteins and identified TFs showed differentially altered abundance only under heat stress, and could potentially be regulated directly or indirectly by miR156 (Fig. 5). In summary, the results from this study have increased our understanding of miR156 and miR156-mediated regulation that could result in potential tangible targets for practical applications in alfalfa and related legume species to address abiotic stress limitations to agricultural productivity. Transcription factors play an important role in regulating the molecular response of plants to stress. Detection of expression changes by transcriptome sequencing analysis in alfalfa could identify genes and transcription factors involved in heat stress tolerance. Therefore, future research should focus on combining physiology with the transcriptome, metabolome, and proteome under the influence of miR156 to provide better insights into the crosstalk between different functional pathways and the regulatory mechanisms controlled by miR156 for heat tolerance in plants.

## Methods

### Plant material, experimental design, and heat treatment

A miR156 overexpressing (miR156OE) alfalfa genotype (A8) was generated by our group in a previous study [21]. Rooted stem cuttings were made from A8 and empty vector (EV) control plant. Stem cuttings were transferred to 5 1/2″ standard pots containing homogenized PRO-MIX® BX soil. Emerging plants were then grown on the bench in the greenhouse under a 16-h light/ 8-h dark regime and watered twice weekly. A randomized experiment was designed and a heat stress trial was started on two-month-old plants of EV and A8, which were randomly assigned to non-stressed control or heat treatments. Heat stress treatment was set up as described in our published study [35]. A minimum of three plants from each genotype were completely randomized in one growth cabinet for heat treatment after watering them to field capacity, whereas the same number of plants were kept in the greenhouse for the non-stress control experiment. Growth cabinet temperature for the heat stress treatment was set to 40 °C and the same photoperiod and light intensity were used as in the greenhouse. Whole plant shoot (above-ground portion) tissues from at least three plants from each of A8 and EV under non-stressed control (22 °C), and after 24 h of heat treatment (40 °C), were collected in separate falcon tubes which were immediately frozen in liquid nitrogen and stored at − 80 °C for further analysis. Frozen shoot tissues from each plant were ground separately into a fine powder with mortal and pestle in liquid nitrogen. Samples required for protein extraction, and proline and antioxidant assays were obtained from this fine powder mixture.

### Proline and antioxidant assays

Proline and antioxidant assays were performed on at least three plants (three biological replicates) from each genotype (EV and A8) obtained from each non-stressed control and heat stressed conditions. Proline measurements were conducted by following a previously described protocol [68]. Briefly, 0.5 g leaf samples were homogenized in 10 ml of 3% sulfosalicylic acid followed by filtration through Whatman #2 filter paper. Subsequently, 2 mL of filtrate was mixed with an equal volume of acid-ninhydrin and glacial acetic acid and reacted at 100 °C for 1 h. The reaction was terminated by cooling on ice, followed by extraction with toluene. The proline content was determined by measuring the absorbance of the supernatant at 520 nm using a plate reader (BioTek, Synergy 2, Winooski, VT) and comparing the values to a standard curve as described in previously published study [26].

For antioxidant measurements, approximately 100 mg of samples were used by following the protocol of an antioxidant assay kit (Sigma-Aldrich, Oakville ON; catalogue number CS0790). Trolox standards were prepared following the protocol provided in the kit. Assays were conducted in a 96-well plate by reading the endpoint absorbance at 405 nm in the plate reader (BioTek). Antioxidant concentration in the samples was calculated by comparing it to Trolox standard curve [26].

### Protein extraction, lysis, and digestion

Protein extraction was performed as described in Marx et al. (2016) [62]. Briefly, leaf samples from three non-stressed control and three heat stressed plants from each of the EV and A8 genotypes were ground into a powder using a mortal and a pestle in liquid nitrogen. The extraction buffer (290 mM sucrose, 250 mM TRIS (pH 7.6), 25 mM EDTA (pH 8.0), 10 mM KCl, 25 mM NaF, 50 mM Na pyrophosphate, 1 mM ammonium molybdate, 1 mM PMSF, 1 μg/mL leupeptin, 1 μg/mL pepstatin, 1 μg/mL aprotinin) [69], and five times volume was added to each sample. The sample mix was then subjected to 3 min probe sonication followed by filtration through Miracloth. Chloroform/methanol was used to precipitate the protein from the sample extract. The obtained protein pellet was washed with 80% cold acetone, air dried for 1 h and used in subsequent steps. The protein pellet was dissolved in lysis buffer (8 M urea, 50 mM Tris-HCl (pH 8), 30 mM NaCl, 1 mM CaCl_2_, 20 mM sodium butyrate, 10 mM nicotinamide, a mini EDTA-free protease inhibitor, and phosSTOP phosphatase inhibitor), followed by additional sonication. The protein content was measured using a bicinchoninic acid (BCA) assay (Thermo Fisher Scientific, Waltham, MA). Subsequently, 75 μg protein were reduced with dithiothreitol and alkylated with 15 mM iodoacetamide. Protein digestion was carried out in two steps. First, LysC (Thermo Fisher Scientific) was added followed by incubation at 37 °C for 2.5 h. Second, samples were diluted using 50 mM Tris and 5 mM CaCl_2_. Mass Spec-grade trypsin protease (Thermo Fisher Scientific) was then added to the mixture, which was incubated overnight at ambient temperature. The digestion reaction was quenched by bringing pH ~ 2 using trifluoroacetic acid, immediately desalted using Waters Oasis HLB (1 ml, 30 mg sorbent), and the eluent was dried by vacuum centrifugation. Samples were reconstituted in 75 μL of 0.1% formic acid and transferred to an HPLC vial.

The peptide digests were separated on an Easy-nLC 1000 nano-flow HPLC system equipped with a 2 cm Acclaim C18 PepMap™ trap column and a 75 μm × 25 cm Acclaim C18 PepMap™ analytical column (Thermo Scientific). The flow rate was held at 300 nL min^− 1^ throughout the run and 10 μL of the digest was injected. The mobile phase A (97%) (LC/MS Optima water, 0.1% formic acid) was first decreased to 90% over 4 min. Peptides were then eluted with a linear gradient of 10 to 40% mobile phase B (LC/MS Optima acetonitrile, 0.1% formic acid) over 150 min, followed by 40–90% over 10 min, and maintained constant for an additional 10 min. Each sample was then analyzed using a top 10, data-dependent acquisition method in the mass range of *m/z* 300–2000 using a Thermo Q-Exactive Orbitrap mass spectrometer coupled to an Agilent 1290 HPLC system. The nanospray voltage was set at 2.4 kV, capillary temperature at 275 °C, and the S-lens radio frequency (RF) level at 70. The full scan was acquired at 70,000 resolution with an automatic gain control (AGC) of 1 × 10^6^ and a maximum injection time (IT) of 250 msec. The MS/MS scans were acquired at 17,500 resolution, AGC of 5 × 10^5^, maximum IT of 110 msec, intensity threshold of 1 × 10^5^, normalized collision energy of 27 and an isolation window of 1.7 *m/z*. Unassigned, singly charged, and > 4 charged peptides were not selected for MS/MS, and a 30 s dynamic exclusion was used.

### LC-MS/MS data analysis

#### Data analysis with MaxQuant

Alfalfa (*Medicago sativa*) genome has not been sequenced and therefore we used its close relative *Medicago truncatula* for analysis. The Thermo® raw files were searched against the related species *Medicago truncatula* protein sequence database (Uniprot UP000002051, accessed December 21st 2019) with MaxQuant software (1.6.1.0) [41]. Searches were conducted using default precursor mass tolerances (20 ppm for first search and 4.5 for the main search). Trypsin and LysC were selected as enzymes with a maximum of two missed cleavages. Carbamidomethylation was selected as fixed modification, and oxidation of methionine residues as variable modification. The false discovery rate (FDR) for peptide and protein identification was set to 5%, and minimum peptide length was set to seven amino acids. Proteins that were identified by MS/MS in a minimum of three samples biological samples were retained. The MaxLFQ algorithm for label-free quantification (LFQ) and the “Matching between the runs” feature was enabled [41]. The data was imported into Perseus software (http://www.perseus-framework.org) for LFQ comparisons and missing values were imputed with default settings. Only protein groups with measured LFQ values in two of the three sample replicates were retained. The raw mass spectrometry data and the MAXQUANT output files were deposited to the ProteomeXchange Consortium via the PRIDE partner repository with the dataset identifier PXD019560.

#### GO and TF enrichment analysis

Identified proteins were annotated using the Uniprot database [70]. *M. truncatula* GO terms for the selected proteins were searched from the PlantRegMap using GO Term enrichment tool [71, 72], and all the proteins identified with significant altered abundance in this study were used as input to carry out GO enrichment analysis. The enriched GO terms were summarized and plotted following the published REVIGO protocol [37, 73]. The ratios of molecular functions, cellular component and biological process were calculated based on the number of GO terms. TF enrichment was performed by blasting Uniprot IDs of the 91 proteins unique to miR156 against *M. truncatula* TF database. The TFs were identified based on the functional transcription factor binding site feature [72]. Venn diagrams were generated using the Venny tool [74].

### Statistical analysis

GraphPad Prism software (*https://www.graphpad.com/scientific-software/prism/*) was used to statistically test significance of the data. For comparisons between two groups, the Student t-test was used, whereas Perseus software was used to compare protein groups obtained from MaxQuant data.

## Data Availability

The raw mass spectrometry data and the MAXQUANT output files were deposited to the ProteomeXchange Consortium via the PRIDE partner repository with the dataset identifier PXD019560.

## Abbreviations

miR156: microRNA156
miR156OE: microRNA overexpression
FBA: Fructose-bisphosphate aldolase
G-6-PDH: Glucose-6-phosphate 1-dehydrogenase
LFQ: Label-free quantification

## References

[1] Zahran HH (1999). Rhizobium-legume symbiosis and nitrogen fixation under severe conditions and in an arid climate. Microbiol Mol Biol Rev.

[2] Li S, Li F, Wang J, Zhang W, Meng Q, Chen TH, Murata N, Yang X (2011). Glycinebetaine enhances the tolerance of tomato plants to high temperature during germination of seeds and growth of seedlings. Plant Cell Environ.

[3] Vollenweider P, Gunthardt-Goerg MS (2006). Diagnosis of abiotic and biotic stress factors using the visible symptoms in foliage. Environ Pollut.

[4] Battisti DS, Naylor RL (2009). Historical warnings of future food insecurity with unprecedented seasonal heat. Science.

[5] Li W, Wei Z, Qiao Z, Wu Z, Cheng L, Wang Y (2013). Proteomics analysis of alfalfa response to heat stress. PLoS One.

[6] Hu W, Kong H, Guo Y, Zhang Y, Ding Z, Tie W, Yan Y, Huang Q, Peng M, Shi H, Guo A (2016). Comparative physiological and transcriptomic analyses reveal the actions of melatonin in the delay of postharvest physiological deterioration of cassava. Front Plant Sci.

[7] Kamal AHM, Komatsu S (2016). Jasmonic acid induced protein response to biophoton emissions and flooding stress in soybean. J Proteome.

[8] Staudinger C, Mehmeti-Tershani V, Gil-Quintana E, Gonzalez EM, Hofhansl F, Bachmann G, Wienkoop S (2016). Evidence for a rhizobia-induced drought stress response strategy in *Medicago truncatula*. J Proteome.

[9] Ma Q, Kang J, Long R, Zhang T, Xiong J, Zhang K, Wang T, Yang Q, Sun Y (2017). Comparative proteomic analysis of alfalfa revealed new salt and drought stress-related factors involved in seed germination. Mol Biol Rep.

[10] Zhang C, Shi S (2018). Physiological and proteomic responses of contrasting alfalfa (*Medicago sativa* L.) varieties to PEG-induced osmotic stress. Front Plant Sci.

[11] Xiong J, Sun Y, Yang Q, Tian H, Zhang H, Liu Y, Chen M (2017). Proteomic analysis of early salt stress responsive proteins in alfalfa roots and shoots. Proteome Sci.

[12] Chen L, Chen Q, Zhu Y, Hou L, Mao P (2016). Proteomic identification of differentially expressed proteins during alfalfa (*Medicago sativa* L.) flower development. Front Plant Sci.

[13] Heazlewood JL (2011). The green proteome: challenges in plant proteomics. Front Plant Sci.

[14] Carroll AW, Joshi HJ, Heazlewood JL (2013). Managing the green proteomes for the next decade of plant research. Front Plant Sci.

[15] Lee DG, Ahsan N, Lee SH, Kang KY, Bahk JD, Lee IJ, Lee BH (2007). A proteomic approach in analyzing heat-responsive proteins in rice leaves. Proteomics..

[16] Zou J, Liu C, Chen X (2011). Proteomics of rice in response to heat stress and advances in genetic engineering for heat tolerance in rice. Plant Cell Rep.

[17] Wei YJ, Huang YX, Shen Y, Cui CJ, Zhang XL, Zhang H, Hu SS (2009). Proteomic analysis reveals significant elevation of heat shock protein 70 in patients with chronic heart failure due to arrhythmogenic right ventricular cardiomyopathy. Mol Cell Biochem.

[18] Jones-Rhoades MW, Bartel DP, Bartel B (2006). MicroRNAs and their regulatory roles in plants. Annu Rev Plant Biol.

[19] Hannoufa A, Matthews C, Feyissa BA, Gruber MY, Arshad M (2018). Progress toward deep sequencing-based discovery of stress-related microRNA in plants and available bioinformatics tools. Progress in Botany 81.

[20] Axtell MJ (2013). Classification and comparison of small RNAs from plants. Annu Rev Plant Biol.

[21] Aung B, Gruber MY, Amyot L, Omari K, Bertrand A, Hannoufa A (2015). MicroRNA156 as a promising tool for alfalfa improvement. Plant Biotechnol J.

[22] Aung B, Gruber MY, Amyot L, Omari K, Bertrand A, Hannoufa A (2015). Ectopic expression of LjmiR156 delays flowering, enhances shoot branching, and improves forage quality in alfalfa. Plant Biotechnol Rep.

[23] Arshad M, Gruber M, Hannoufa A (2018). Transcriptome analysis of microRNA156 overexpression alfalfa roots under drought stress. Sci Rep.

[24] Cardon G, Höhmann S, Klein J, Nettesheim K, Saedler H, Huijser P (1999). Molecular characterisation of the Arabidopsis SBP-box genes. Gene..

[25] Gao R, Gruber MY, Amyot L, Hannoufa A (2018). SPL13 regulates shoot branching and flowering time in *Medicago sativa*. Plant Mol Biol.

[26] Arshad M, Feyissa BA, Amyot L, Aung B, Hannoufa A (2017). MicroRNA156 improves drought stress tolerance in alfalfa (*Medicago sativa*) by silencing *SPL13*. Plant Sci.

[27] Arshad M, Gruber MY, Wall K, Hannoufa A (2017). An insight into microRNA156 role in salinity stress responses of alfalfa. Front Plant Sci.

[28] Claussen W (2005). Proline as a measure of stress in tomato plants. Plant Sci.

[29] Rivero R, Ruiz M, Romero LM (2004). Importance of N source on heat stress tolerance due to the accumulation of proline and quaternary ammonium compounds in tomato plants. Plant Biol.

[30] Hamilton EW (2001). Mitochondrial adaptations to NaCl. Complex I is protected by anti-oxidants and small heat shock proteins, whereas complex II is protected by proline and betaine. Plant Physiol.

[31] Tonhati R, Mello SC, Momesso P, Pedroso RM (2020). L-proline alleviates heat stress of tomato plants grown under protected environment. Sci Hortic.

[32] Abdula SE, Lee HJ, Ryu H, Kang KK, Nou I, Sorrells ME, Cho YG (2016). Overexpression of BrCIPK1 gene enhances abiotic stress tolerance by increasing proline biosynthesis in rice. Plant Mol Biol Rep.

[33] Kaushal N, Gupta K, Bhandhari K, Kumar S, Thakur P, Nayyar H (2011). Proline induces heat tolerance in chickpea (*Cicer arietinum* L.) plants by protecting vital enzymes of carbon and antioxidative metabolism. Physiol Mol Biol Plants.

[34] Oukarroum A, Madidi ES, Strasser RJ (2012). Exogenous glycine betaine and proline play a protective role in heat-stressed barley leaves (*Hordeum vulgare* L.): a chlorophyll a fluorescence study. Plant Biosystems-An Int J Deal Aspects Plant Biol.

[35] Matthews C, Arshad M, Hannoufa A (2019). Alfalfa response to heat stress is modulated by microRNA156. Physiol Plant.

[36] Ashraf M (2010). Inducing drought tolerance in plants: recent advances. Biotechnol Adv.

[37] Gao R, Austin RS, Amyot L, Hannoufa A. Comparative transcriptome investigation of global gene expression changes caused by miR156 overexpression in Medicago sativa. BMC Genomics. 2016;17. 10.1186/s12864-016-3014-6. 10.1186/s12864-016-3014-6 PMC4992203 27542359

[38] Feyissa BA, Arshad M, Gruber MY, Kohalmi SE, Hannoufa A (2019). The interplay between miR156/SPL13 and DFR/WD40–1 regulate drought tolerance in alfalfa. BMC Plant Biol.

[39] Teixeira EI, Fischer G, Van Velthuizen H, Walter C, Ewert F (2013). Global hot-spots of heat stress on agricultural crops due to climate change. Agric Forest Meteorol.

[40] Wahid A, Gelani S, Ashraf M, Foolad MR (2007). Heat tolerance in plants: an overview. Environ Exp Bot.

[41] Cox J, Hein MY, Luber CA, Paron I, Nagaraj N, Mann M (2014). Accurate proteome-wide label-free quantification by delayed normalization and maximal peptide ratio extraction, termed MaxLFQ. Mol Cell Proteomics.

[42] Gilroy S, Bialasek M, Suzuki N, Gorecka M, Devireddy AR, Karpinski S, Mittler R (2016). ROS, calcium, and electric signals: key mediators of rapid systemic signaling in plants. Plant Physiol.

[43] Anjum NA, Aref IM, Duarte AC, Pereira E, Ahmad I, Iqbal M. Glutathione and proline can coordinately make plants withstand the joint attack of metal(loid) and salinity stresses. Front Plant Sci. 2014;5. 10.3389/fpls.2014.00662. 10.3389/fpls.2014.00662 PMC4240066 25484889

[44] Cui LG, Shan JX, Shi M, Gao JP, Lin HX (2014). The miR156-SPL9-DFR pathway coordinates the relationship between development and abiotic stress tolerance in plants. Plant J.

[45] Li N, Zhang S, Liang YJ, Qi YH, Chen J, Zhu WN, Zhang LS (2018). Label-free quantitative proteomic analysis of drought stress-responsive late embryogenesis abundant proteins in the seedling leaves of two wheat (*Triticum aestivum* L.) genotypes. J Proteome.

[46] Das A, Eldakak M, Paudel B, Kim DW, Hemmati H, Basu C, Rohila JS. Leaf proteome analysis reveals prospective drought and heat stress response mechanisms in soybean. Biomed Res Int. 2016. 10.1155/2016/6021047. 10.1155/2016/6021047 PMC4808539 27034942

[47] Sah SK, Reddy KR, Li JX. Abscisic acid and abiotic stress tolerance in crop plants. Front Plant Sci. 2016;7. 10.3389/fpls.2016.00571. 10.3389/fpls.2016.00571 PMC4855980 27200044

[48] Chaves MM, Flexas J, Pinheiro C (2009). Photosynthesis under drought and salt stress: regulation mechanisms from whole plant to cell. Ann Bot.

[49] Lawlor DW, Tezara W (2009). Causes of decreased photosynthetic rate and metabolic capacity in water-deficient leaf cells: a critical evaluation of mechanisms and integration of processes. Ann Bot.

[50] Baena-Gonzalez E, Rolland F, Thevelein JM, Sheen J (2007). A central integrator of transcription networks in plant stress and energy signalling. Nature.

[51] Yin Y, Li SM, Liao WQ, Lu QT, Wen XG, Lu CM (2010). Photosystem II photochemistry, photoinhibition, and the xanthophyll cycle in heat-stressed rice leaves. J Plant Physiol.

[52] Liu GT, Ma L, Duan W, Wang BC, Li JH, Xu HG, Yan XQ, Yan BF, Li SH, Wang LJ (2014). Differential proteomic analysis of grapevine leaves by iTRAQ reveals responses to heat stress and subsequent recovery. BMC Plant Biol.

[53] Rinalducci S, Egidi MG, Karimzadeh G, Jazii FR, Zolla L (2011). Proteomic analysis of a spring wheat cultivar in response to prolonged cold stress. Electrophoresis..

[54] Oukarroum A, Schansker G, Strasser RJ (2009). Drought stress effects on photosystem I content and photosystem II thermotolerance analyzed using Chl a fluorescence kinetics in barley varieties differing in their drought tolerance. Physiol Plant.

[55] Aranjuelo I, Molero G, Erice G, Avice JC, Nogues S (2011). Plant physiology and proteomics reveals the leaf response to drought in alfalfa (*Medicago sativa L.*). J Exp Bot.

[56] Sahu AC, Sahoo SK, Sahoo N (2001). NaCl-stress induced alteration in glutamine synthetase activity in excised senescing leaves of a salt-sensitive and a salt-tolerant rice cultivar in light and darkness. Plant Growth Regul.

[57] el-Khatib RT, Hamerlynck EP, Gallardo F, Kirby EG (2004). Transgenic poplar characterized by ectopic expression of a pine cytosolic glutamine synthetase gene exhibits enhanced tolerance to water stress. Tree Physiol.

[58] de las Mercedes Dana M, Pintor-Toro JA, Cubero B (2006). Transgenic tobacco plants overexpressing chitinases of fungal origin show enhanced resistance to biotic and abiotic stress agents. Plant Physiol.

[59] Sarwat M, Naqvi AR (2013). Heterologous expression of rice calnexin (OsCNX) confers drought tolerance in *Nicotiana tabacum*. Molecular Biol Rep.

[60] Ban Q, Jiao J, He Y, Jin M, Rao J (2020). Ectopic expression of the persimmon β-galactosidase gene DkGAL2 promotes leaf growth, delays dark-induced senescence and enhances tolerance to abiotic stress in Arabidopsis. Sci Hortic.

[61] Pennycooke JC, Jones ML, Stushnoff C (2003). Down-regulating α-galactosidase enhances freezing tolerance in transgenic petunia. Plant Physiol.

[62] Bita CE, Gerats T. Plant tolerance to high temperature in a changing environment: scientific fundamentals and production of heat stress-tolerant crops. Front Plant Sci. 2013;4. 10.3389/fpls.2013.00273. 10.3389/fpls.2013.00273 PMC3728475 23914193

[63] Jacob P, Hirt H, Bendahmane A (2017). The heat-shock protein/chaperone network and multiple stress resistance. Plant Biotechnol J.

[64] Heckathorn SA, Downs CA, Coleman JS (1999). Small heat shock proteins protect electron transport in chloroplasts and mitochondria during stress. Am Zool.

[65] Downs CA, Coleman JS, Heckathorn SA (1999). The chloroplast 22-Ku heat-shock protein: A lumenal protein that associates with the oxygen evolving complex and protects photosystem II during heat stress. J Plant Physiol.

[66] Kim KH, Alam I, Kim YG, Sharmin SA, Lee KW, Lee SH, Lee BH (2012). Overexpression of a chloroplast-localized small heat shock protein OsHSP26 confers enhanced tolerance against oxidative and heat stresses in tall fescue. Biotechnol Lett.

[67] Liu Y, Wu R, Wan Q, Xie G, Bi Y (2007). Glucose-6-phosphate dehydrogenase plays a pivotal role in nitric oxide-involved defense against oxidative stress under salt stress in red kidney bean roots. Plant Cell Physiol.

[68] Abraham E, Hourton-Cabassa C, Erdei L, Szabados L (2010). Methods for determination of proline in plants. Methods Mol Biol.

[69] Marx H, Minogue CE, Jayaraman D, Richards AL, Kwiecien NW, Siahpirani AF, Rajasekar S, Maeda J, Garcia K, Del Valle-Echevarria AR, Volkening JD, Westphall MS, Roy S, Sussman MR, Ane JM, Coon JJ (2016). A proteomic atlas of the legume *Medicago truncatula* and its nitrogen-fixing endosymbiont Sinorhizobium meliloti. Nat Biotechnol.

[70] Pundir S, Martin MJ, O'Donovan C (2017). UniProt protein knowledgebase. Methods Mol Biol.

[71] Jin J, Tian F, Yang DC, Meng YQ, Kong L, Luo J, Gao G (2017). PlantTFDB 4.0: toward a central hub for transcription factors and regulatory interactions in plants. Nucleic Acids Res.

[72] Tian F, Yang D, Meng Y, Jin J, Gao G (2020). PlantRegMap: charting functional regulator maps in plants. Nucleic Acid Res.

[73] Supek F, Bosnjak M, Skunca N, Smuc T (2011). REVIGO summarizes and visualizes long lists of gene ontology terms. PLoS One.

[74] Oliveros JC (2007). “Venny”. An interactive tool for comparing lists with Venn Diagrams.

